# Factors Affecting COVID-19 Testing Behaviours Among the Population in South Western Nigeria

**DOI:** 10.3389/ijph.2022.1604993

**Published:** 2022-10-06

**Authors:** Olufemi Samuel Amoo, Bosun Tijani, Tochukwu Ifeanyi Onuigbo, Joy Isioma Oraegbu, Dorcas Njeri Kareithi, Josephine Chioma Obi, Esther Temilade Adeniji, Adenike Aderonke Dosunmu, Steven Karera, Temi Filani, Temidayo Akinreni, Emmanuella Ezike, Kehinde Owoseni, Rosemary Ajuma Audu, Babatunde Lawal Salako

**Affiliations:** ^1^ Nigerian Institute of Medical Research (NIMR), Lagos, Nigeria; ^2^ Co-creation Hub, Lagos, Nigeria

**Keywords:** testing, knowledge, willingness, behaviours, factors

## Abstract

**Objectives:** The objective of this study was to assess the factors affecting testing behaviours amongst the population in Ondo and Lagos States.

**Methods:** A cross-sectional study involving 704 individuals who were considered eligible for COVID-19 testing in 4 local governments in Lagos (307) and Ondo (397) states in Nigeria, was conducted from April-June 2021. Respondents were selected using simple random sampling. A close-ended questionnaire was administered using a digital survey platform known as SurveyCTO. Data were analyzed using R 4.1.0.

**Results:** In Lagos state, 52.4% were females, 47.2% were males while in Ondo, 55.2% were females, 44.6% were male. Chi-square tests of association revealed that socio demographic factors significantly associated with testing patterns was education level in Lagos, and none in Ondo. Testing behavior associated with testing patterns included awareness of nearby COVID-19 testing centers, internet access, knowledge of preexisting conditions and having another member of the family testing positive at 5% significance level.

**Conclusion:** Knowledge of pre-existing conditions, knowledge of COVID-19 symptoms, and knowing where to go when having symptoms were significantly associated with testing and willingness to test.

## Introduction

When the Coronavirus Disease 2019 (COVID-19) became a pandemic in March 2020, the World Health Organization (WHO) worked with other global partners and country health systems to roll out the COVID-19 treatment guidelines, protocols, standard operating procedures (SOPs) and best practices [1]. The rollout was done at the national level, with each stratum of government mandated to ensure that they are adhered to within the country. The guidelines cover a spectrum of topics, including management of cases, testing procedures and sensitization. However, implementation of these guidelines at country level differs, and is affected by several factors that are related to care- seeking behaviour of citizens [2, 3].

In order to ensure the successful management of COVID-19, the compliance of individuals to stipulated guidelines for prevention and control is greatly determined by their knowledge, attitudes and practices [4]. Nigeria has increased its testing capacity to facilitate extensive testing, with an average testing rate of 6.4% as of 2nd February 2022 [5]. The effectiveness of mass and rapid testing depends not only on how the tests are conducted, but the willingness of citizens to be tested among other factors. Voluntary random testing is highly recommended and has been found to be effective in some countries [6]. This, however, has been mentioned to lead to systematic selection bias because individuals who prefer to learn about their health status are the most likely to seek voluntary testing [7]. Several factors could affect or influence this preference. Tijani et al. [8] reported in their study that distance to facility, preexisting health conditions, knowledge of COVID-19 symptoms, previous healthcare experience, type and frequency of communication influence healthcare seeking behavior of Nigerians, specifically during the COVID-19 pandemic. It should be noted, however, that factors that could influence testing behaviours are vast and could vary based on environment, knowledge of the people of concern as well as even cultural practices of certain areas [9].

In Nigeria, Lagos and Ondo represent two states with different archetypes. Both states have reported COVID-19 cases since the onset of the pandemic 98,693 cases in Lagos and 5,173 in Ondo state as of 16th February 2022 [5]. Despite the desire by the national and state governments to ensure a more equitable distribution of health resources, glaring disparities in healthcare delivery are still evident. This is anticipated to affect the proportion of people who tested for COVID-19. In order to ascertain the factors affecting testing behaviours in south western Nigeria, there is therefore a need to assess these factors amongst citizens of Ondo and Lagos States.

## Methods

### Study Design

A cross-sectional study involving people who were considered eligible for COVID-19 testing in Lagos and Ondo states in Nigeria was conducted. The study took place in 4 local governments in Lagos (Shomolu, Ikorodu, Ibeju-Lekki and Surulere) and in Ondo (Ifedore, Akoko, Akure North and Akure South Local government) states between April and June 2021. The respondents were selected using simple random sampling where every individual in the target population has an equal chance of being selected.

## Questionnaire Design and Pretest

### Data Collection

Data was collected using two methods:

• **Desk Review:** COVID-19 protocols and practices from the two states were reviewed from the ministry of health and other government platforms using desk review. A template was provided to collect specific information regarding the COVID-19 protocols and practices.
• **A closed-ended questionnaire**: A close-ended questionnaire was used to collect information related to testing behaviours among the citizens of Ondo and Lagos states. The survey was converted to a digital survey using SurveyCTO. Trained data collectors were used to administer the questionnaire to the randomly selected participants.
• **Questionnaire pretest:** The questionnaire was reviewed by experts at NIMR, CcHUB Research and CcHUB public health units and revised based on their comments. Subsequently, it was pilot-tested on 10 participants in Ondo and 10 participants in Lagos, who were excluded in the main study), to check for its applicability and clarity before commencement of the study. All the necessary modifications were done based on the outcome of the pilot study.

### Data Transmission

Data from the closed-ended questionnaire was automatically downloaded from a secure server, and transmitted real time by SurveyCTO. High frequency checks were conducted, with variables like age, location and other demographics, being compared with data in the Demographic Health Survey (DHS) results for validation.

### Data Management and Analysis

Multiple response questions in the “Testing and Knowledge” section were transformed to binary variables. In addition, all testing and knowledge related questions were collapsed to two categories. The outcome variable was also generated from two questions i.e., “Have you tested for COVID-19” and “Are you interested in testing for COVID-19?” as shown on the Table 1.

**TABLE 1:** New variables definition, Nigeria, April-June 2021.

Variable	Levels	Criteria
Covid testing	0 = Not tested, not interested	Whether a respondent has been tested, and if not, if they are interested
	1 = Not tested, interested in testing	
	2 = Tested	
Knowledge on Pre-existing conditions	1 = Knowledgeable	Respondent classified as knowledgeable if they know any two pre-conditions
	2 = Not Knowledgeable	
Knowledge on Symptoms	1 = Knowledgeable	Respondent are classified as knowledgeable if they know any five of the symptoms otherwise they are Not knowledgeable
	2 = Not knowledgeable	
Knowledge on where to go when having symptoms	1 = Knowledgeable	Respondent are classified as knowledgeable if they know any two of the places
	2 = Not knowledgeable	
Knowledge on how to react when having symptoms	1 = Knowledgeable	Respondents are classified as knowledgeable if they know any one of the reactions to symptoms
	2 = Not knowledgeable	

Data was analyzed using frequency tables, cross tabulations, graphs, chi-square and multinomial logistic regression. The multinomial logistic regression model fitted was:

$$\ln\;\left(\frac{p\left(category\right)}{p\left(base\;category\right)}\right)=b_{0j}+b_{ji}x_{i}$$

Where: *Category of patients* = 1) Respondents who have not tested for COVID-19 but are interested in testing, 2) Respondents who have tested for COVID-19.

*Base category* = Respondents who have not tested for COVID-19 and are not interested in testing.

$b_{0}=intercept\;when\;comparing\;base\;category\;and\;category\;j$

$b_{ij}=the\;coefficient\;of\;predictor\;X_{i}$

$X_{i}=predictor\;variable$

In this model, we sought to assess what category participants would fall into, given their demographic characteristics, their knowledge levels, their behavior and other characteristics.

All descriptive and inferential analyses were based on the above three (3) groups. All data were analyzed using R 4.1.0.

## Results

### Socio-Demographic Characteristics

A total of 773 individuals were approached to partake in this study. Only 704 (91.1%) of which 307 (43.6%) participants were from Lagos and 397 (56.4%) participants from Ondo state consented to answer the questionnaire. Furthermore, in Lagos, 52.4% (*n* = 161) were females while 47.2% (*n* = 145) were males and 0.3% (*n* = 1) preferred not to say their gender. In Ondo, 55.2% (*n* = 219) were females while 44.6% (*n* = 177) were males and 0.2% (*n* = 1) preferred not to say their gender. More than half of the participants 58.2% (*n* = 410) were between 20 and 39 years old and majority of the participants 95.2% (*n* = 670) had at least completed the Senior Secondary level of Education (Table 2).

**TABLE 2:** Demographic characteristics, Nigeria, April-June 2021.

Variable	Lagos (*n* = 307)	Ondo (*n* = 397)	Chi-square (df)
Gender			
Female	52.4% (*n* = 161)	55.2% (*n* = 219)	0.55 (df = 2)
Male	47.2% (*n* = 145)	44.6% (*n* = 177)	
Not specified	0.3% (*n* = 1)	0.2% (*n* = 1)	
Marital status			
Never married	53.8% (*n* = 165)	43.8% (*n* = 174)	11.25 (df = 3)**
Married	41.0% (*n* = 126)	53.2% (*n* = 211)	
Widowed	3.6% (*n* = 11)	1.8% (*n* = 7)	
Divorced	1.6% (*n* = 5)	1.2% (*n* = 5)	
Age group			
19 and below	14.7% (*n* = 45)	12.1% (*n* = 48)	7.52 (df = 7)
20–29	33.6% (*n* = 103)	29.7% (*n* = 118)	
30–39	28.3% (*n* = 87)	25.7% (*n* = 102)	
40–49	9.5% (*n* = 29)	14.4% (*n* = 57)	
50–59	8.5% (*n* = 26)	10.3% (*n* = 41)	
60–69	2.9% (*n* = 9)	4.0% (*n* = 16)	
70–79	1.6% (*n* = 5)	2.5% (*n* = 10)	
80 and above	0.9% (*n* = 3)	1.3% (*n* = 5)	
Education			
Never attended school/Kindergarten	0.7% (*n* = 2)	1.3% (*n* = 5)	50.06 (df = 6)***
Primary	3.9% (*n* = 12)	1.3% (*n* = 5)	
Junior Secondary	1.9% (*n* = 6)	1.0% (*n* = 4)	
Senior Secondary	33.2% (*n* = 102)	14.6% (*n* = 58)	
Vocational	18.9% (*n* = 58)	20.1% (*n* = 80)	
Tertiary Institution	41.4% (*n* = 127)	61.7% (*n* = 245)	

* *p* < 0.05. **p < 0.01. ***`*p* < 0.001.

Only 10.7% (*n* = 75) from the two states reported to have tested for COVID-19 while the remaining 89.3% (*n* = 629) reported not tested for COVID-19. Only 15.6% (*n* = 48) of the participants from Lagos have tested while only 6.8% (*n* = 27) from Ondo State have tested for COVID-19. Data from Lagos showed that 14.3% (*n* = 23) of female participants and 17.2% (*n* = 25) of males had tested for COVID-19; while in Ondo state, approximately 6.8% (*n* = 15) of females and males 6.8% (*n* = 12) had tested for COVID-19 (Table 3).

**TABLE 3:** Testing pattern, Nigeria, April-June 2021.

Variable	Lagos (*n* = 307)	Ondo (*n* = 397)	Chi-square
Family member tested positive			
Yes	17.6%(*n* = 54)	3.3%(*n* = 13)	45.42***
No	64.2%(*n* = 197)	81.6(*n* = 324)	
Not sure	18.2%(*n* = 56)	15.1%(*n* = 60)	
Have you tested for COVID-19			
Yes	15.6%(*n* = 48)	6.8%(*n* = 27)	14.19***
No	84.4% (*n* = 259)	93.2%(*n* = 370)	
Aware of testing centers			
No	63.5%(*n* = 195)	60.20%(*n* = 239)	0.80
Yes	36.5%(*n* = 112)	39.8%(*n* = 158)	
Distance from testing center			
Less than 1 Km	1.0%(*n* = 3)	3.5%(*n* = 14)	21.12***
Between 1 km and 5 km	10.4%(*n* = 32)	13.1%(*n* = 52)	
Between 5 and 10 km	16.3%(*n* = 50)	8.1%(*n* = 32)	
More than 10 km	8.8%(*n* = 27)	15.1%(*n* = 60)	
Not Applicable	63.5%(*n* = 195)	60.2%(*n* = 239)	
Time to reach the testing center			
1–2 h	2.0%(*n* = 6)	4.5%(*n* = 18)	14.82**
15 min or less	11.7%(*n* = 36)	7.3%(*n* = 29)	
16–30 min	12.7%(*n* = 39)	11.3%(*n* = 45)	
36–45 min	5.2%(*n* = 16)	9.3%(*n* = 37)	
46 to 1 h	1.6%(*n* = 5)	4.3%(*n* = 17)	
More than 2 h	3.3%(*n* = 10)	3.1%(*n* = 12)	
Not Applicable	63.5%(*n* = 195)	60.2%(*n* = 239)	

* *p* < 0.05 **p < 0.01***p < 0.001.

### Testing Pattern and Behavior

Statistically significant number of participants from Lagos indicated that they have members of their family 17.6% (*n* = 54) who tested positive to COVID-19 with about 3.3% (*n* = 13) in Ondo state (Table 3). Participants in Ondo state were more aware (39.8%) of a nearby testing center than those in Lagos (36.5%). With regards to the distance and time taken to reach a testing center, there is a statistical difference between the two states (*p* = 0.000). In Lagos, 16.3% (*n* = 50) reported a distance of 5–10 km to the nearest testing center while in Ondo, 15.1% (*n* = 60) reported a distance of more than 10 km to the nearest testing center*.* Participants from both states; Lagos:12.7% (*n* = 39), Ondo:11.3% (*n* = 45) would travel between 16 and 30 min to get to a testing center. The association between Lagos and Ondo participants in relation to Family members testing positive to COVID-19 and having tested for COVID-19 were statistically significant at *p* < 0.000 (Table 3).

### Testing and Knowledge of Predisposing Factors

On the knowledge of testing, 70.7% (*n* = 53) of those who tested in Lagos and Ondo States reported that they knew pre-conditions that can complicate COVID-19 infection. Moreover, 72.9% (*n* = 35) of those who tested in Lagos knew about COVID-19 symptoms with 59.3% (*n* = 16) reporting the same in Ondo state. A chi-square test of independence was performed to examine the association between knowledge variables (pre-conditions, Symptoms, where to go) and testing behaviors, the relation between these variables showed statistically significant association between knowledge and state (Table 4).

**TABLE 4:** Tests of Association results, Nigeria, April-June 2021.

Variable	Lagos				Ondo			
	Tested	Not tested, interested in testing	Not tested, not interested	Chisq (df)	Tested	Not tested, interested in testing	Not tested, not interested	Chisq (df)
Age group				19.32(14)				19.57(14)
19 and below	11.11% (*n* = 5)	6.67% (*n* = 3)	82.22% (*n* = 37)		2.08% (*n* = 1)	0% (*n* = 0)	97.92% (*n* = 47)	
20–29	12.62% (*n* = 13)	10.68% (*n* = 11)	76.7% (*n* = 79)		5.08% (*n* = 6)	4.24% (*n* = 5)	90.68% (*n* = 107)	
30–39	20.69% (*n* = 18)	14.94% (*n* = 13)	64.37% (*n* = 56)		6.86% (*n* = 7)	8.82% (*n* = 9)	84.31% (*n* = 86)	
40–49	31.03% (*n* = 9)	3.45% (*n* = 1)	65.52% (*n* = 19)		8.77% (*n* = 5)	1.75% (*n* = 1)	89.47% (*n* = 51)	
50–59	7.69% (*n* = 2)	3.85% (*n* = 1)	88.46% (*n* = 23)		9.76% (*n* = 4)	0% (*n* = 0)	90.24% (*n* = 37)	
60–69	11.11% (*n* = 1)	0% (*n* = 0)	88.89% (*n* = 8)		12.5% (*n* = 2)	0% (*n* = 0)	87.5% (*n* = 14)	
70–79	0% (*n* = 0)	20% (*n* = 1)	80% (*n* = 4)		20% (*n* = 2)	0% (*n* = 0)	80% (*n* = 8)	
80 and above	0% (*n* = 0)	0% (*n* = 0)	100% (*n* = 3)		0% (*n* = 0)	0% (*n* = 0)	100% (*n* = 5)	
Gender				4.71(4)				0.14(4)
Male	17.24% (*n* = 25)	13.1% (*n* = 19)	69.66% (*n* = 101)		6.78% (*n* = 12)	3.95% (*n* = 7)	89.27% (*n* = 158)	
Female	14.29% (*n* = 23)	6.83% (*n* = 11)	78.88% (*n* = 127)		6.85% (*n* = 15)	3.65% (*n* = 8)	89.5% (*n* = 196)	
Not specified	0% (*n* = 0)	0% (*n* = 0)	100% (*n* = 1)		0% (*n* = 0)	0% (*n* = 0)	100% (*n* = 1)	
Marital status				6.16(6)				8.12(6)
Never married	12.73% (*n* = 21)	11.52% (*n* = 19)	75.76% (*n* = 125)		3.45% (*n* = 6)	4.6% (*n* = 8)	91.95% (*n* = 160)	
Married	20.63% (*n* = 26)	7.14% (*n* = 9)	72.22% (*n* = 91)		9.48% (*n* = 20)	3.32% (*n* = 7)	87.2% (*n* = 184)	
Divorced	0% (*n* = 0)	20% (*n* = 1)	80% (*n* = 4)		20% (*n* = 1)	0% (*n* = 0)	80% (*n* = 4)	
Widowed	9.09% (*n* = 1)	9.09% (*n* = 1)	81.82% (*n* = 9)		0% (*n* = 0)	0% (*n* = 0)	100% (*n* = 7)	
Education				47.23(10) ***				8.57(12)
Never attended school/Kindergarten	0% (*n* = 0)	0% (*n* = 0)	100% (*n* = 2)		20% (*n* = 1)	0% (*n* = 0)	80% (*n* = 4)	
Primary	0% (*n* = 0)	8.33% (*n* = 1)	91.67% (*n* = 11)		20% (*n* = 1)	0% (*n* = 0)	80% (*n* = 4)	
Junior secondary	33.33% (*n* = 2)	0% (*n* = 0)	66.67% (*n* = 4)		0% (*n* = 0)	0% (*n* = 0)	100% (*n* = 4)	
Senior secondary	5.88% (*n* = 6)	5.88% (*n* = 6)	88.24% (*n* = 90)		1.72% (*n* = 1)	3.45% (*n* = 2)	94.83% (*n* = 55)	
Vocational/technical education	3.45% (*n* = 2)	8.62% (*n* = 5)	87.93% (*n* = 51)		3.75% (*n* = 3)	5% (*n* = 4)	91.25% (*n* = 73)	
Tertiary education	29.92% (*n* = 38)	14.17% (*n* = 18)	55.91% (*n* = 71)		8.57% (*n* = 21)	3.67% (*n* = 9)	87.76% (*n* = 215)	
Family member tested positive				33.83(2)***				104.29(2) ***
No	10.67% (*n* = 27)	8.3% (*n* = 21)	81.03% (*n* = 205)		4.43% (*n* = 17)	3.91% (*n* = 15)	91.67% (*n* = 352)	
Yes	38.89% (*n* = 21)	16.67% (*n* = 9)	44.44% (*n* = 24)		76.92% (*n* = 10)	0% (*n* = 0)	23.08% (*n* = 3)	
Aware of testing centers				94.41(2)***				50.20(2)***
No	1.54% (*n* = 3)	6.67% (*n* = 13)	91.79% (*n* = 179)		0% (*n* = 0)	2.09% (*n* = 5)	97.91% (*n* = 234)	
Yes	40.18% (*n* = 45)	15.18% (*n* = 17)	44.64% (*n* = 50)		17.09% (*n* = 27)	6.33% (*n* = 10)	76.58% (*n* = 121)	
Distance to testing center				1.73(4)				4.98(4)
Less than 5 km	43.75% (*n* = 14)	18.75% (*n* = 6)	37.5% (*n* = 12)		17.31% (*n* = 9)	3.85% (*n* = 2)	78.85% (*n* = 41)	
Between 5 km and 10 km	36% (*n* = 18)	14% (*n* = 7)	50% (*n* = 25)		12.5% (*n* = 4)	15.63% (*n* = 5)	71.88% (*n* = 23)	
More than 10 km	44.44% (*n* = 12)	11.11% (*n* = 3)	44.44% (*n* = 12)		16.67% (*n* = 10)	5% (*n* = 3)	78.33% (*n* = 47)	
Time to reach the testing center				0.64(4)				0.43(4)
Less than an hour	38.67% (*n* = 29)	14.67% (*n* = 11)	46.67% (n = 35)		14.86% (*n* = 11)	8.11% (*n* = 6)	77.03% (*n* = 57)	
About an hour	42.86% (*n* = 9)	19.05% (*n* = 4)	38.1% (n = 8)		16.67% (*n* = 9)	5.56% (*n* = 3)	77.78% (*n* = 42)	
More than an hour	33.33% (*n* = 2)	16.67% (*n* = 1)	50% (*n* = 3)		16.67% (*n* = 3)	5.56% (*n* = 1)	77.78% (*n* = 14)	
Internet access				11.03(4)*				2.93(4)
No access	2.78% (*n* = 1)	5.56% (*n* = 2)	91.67% (*n* = 33)		7.14% (*n* = 3)	2.38% (*n* = 1)	90.48% (*n* = 38)	
At least once a day	18% (*n* = 45)	11.2% (*n* = 28)	70.8% (*n* = 177)		6.91% (*n* = 21)	4.61% (*n* = 14)	88.49% (*n* = 269)	
Rarely	9.52% (*n* = 2)	0% (*n* = 0)	90.48% (*n* = 19)		5.88% (*n* = 3)	0% (*n* = 0)	94.12% (*n* = 48)	
Pre-existing conditions				39.90 (2)***				10.20(2)**
Not Knowledgeable	4.91% (*n* = 8)	6.13% (*n* = 10)	88.96% (*n* = 145)		4.83% (n = 14)	2.76% (n = 8)	92.41% (n = 268)	
Knowledgeable	27.78% (*n* = 40)	13.89% (*n* = 20)	58.33% (*n* = 84)		12.15% (n = 13)	6.54% (n = 7)	81.31% (n = 87)	
Symptoms				33.98(2) ***				2.59(2)
Not Knowledgeable	6.67% (*n* = 13)	12.31% (*n* = 24)	81.03% (*n* = 158)		5.53% (*n* = 11)	5.03% (*n* = 10)	89.45% (*n* = 178)	
Knowledgeable	31.25% (*n* = 35)	5.36% (*n* = 6)	63.39% (*n* = 71)		8.08% (*n* = 16)	2.53% (*n* = 5)	89.39% (*n* = 177)	
Where to go				22.99(2)***				6.55(2)*
Not Knowledgeable	10.04% (*n* = 23)	9.17% (*n* = 21)	80.79% (*n* = 185)		6.67% (*n* = 21)	2.54% (*n* = 8)	90.79% (*n* = 286)	
Knowledgeable	32.05% (*n* = 25)	11.54% (*n* = 9)	56.41% (*n* = 44)		7.32% (*n* = 6)	8.54% (*n* = 7)	84.15% (*n* = 69)	
Response to symptoms				1.11(2)				2.94(2)
Not knowledgeable	16.88% (*n* = 39)	9.52% (*n* = 22)	73.59% (*n* = 170)		7.44% (*n* = 16)	2.33% (*n* = 5)	90.23% (*n* = 194)	
Knowledgeable	11.84% (*n* = 9)	10.53% (*n* = 8)	77.63% (*n* = 59)		6.04% (*n* = 11)	5.49% (*n* = 10)	88.46% (*n* = 161)	
Tech devices (number)				25.88(4)***				10.64(6)
0	5% (*n* = 2)	5% (*n* = 2)	90% (*n* = 36)		10% (*n* = 5)	2% (*n* = 1)	88% (*n* = 44)	
1	13.9% (*n* = 31)	8.52% (*n* = 19)	77.58% (*n* = 173)		4.43% (*n* = 12)	4.06% (*n* = 11)	91.51% (*n* = 248)	
2	34.09% (*n* = 15)	20.45% (*n* = 9)	45.45% (*n* = 20)		14.29% (*n* = 10)	4.29% (*n* = 3)	81.43% (*n* = 57)	
3	0% (*n* = 0)	0% (*n* = 0)	0% (*n* = 0)		0% (*n* = 0)	0% (*n* = 0)	100% (*n* = 6)	
Tech devices (type)				38.12(6)***				11.07(8)
Computer and smartphone	34.09% (*n* = 15)	20.45% (*n* = 9)	45.45% (*n* = 20)		14.29% (*n* = 10)	4.29% (*n* = 3)	81.43% (*n* = 57)	
Smartphone	12% (*n* = 24)	8% (*n* = 16)	80% (*n* = 160)		4.55% (*n* = 12)	4.17% (*n* = 11)	91.29% (*n* = 241)	
None	5% (*n* = 2)	5% (*n* = 2)	90% (*n* = 36)		10% (*n* = 5)	2% (*n* = 1)	88% (*n* = 44)	
Computer/laptop	41.18% (*n* = 7)	17.65% (*n* = 3)	41.18% (*n* = 7)		0% (*n* = 0)	0% (*n* = 0)	100% (*n* = 6)	
Computer and two phones	0% (*n* = 0)	0% (*n* = 0)	0% (*n* = 0)		0% (*n* = 0)	0% (*n* = 0)	100% (*n* = 6)	

*p < 0.05. **p < 0.01. ***p < 0.001.

**FIGURE 1:**
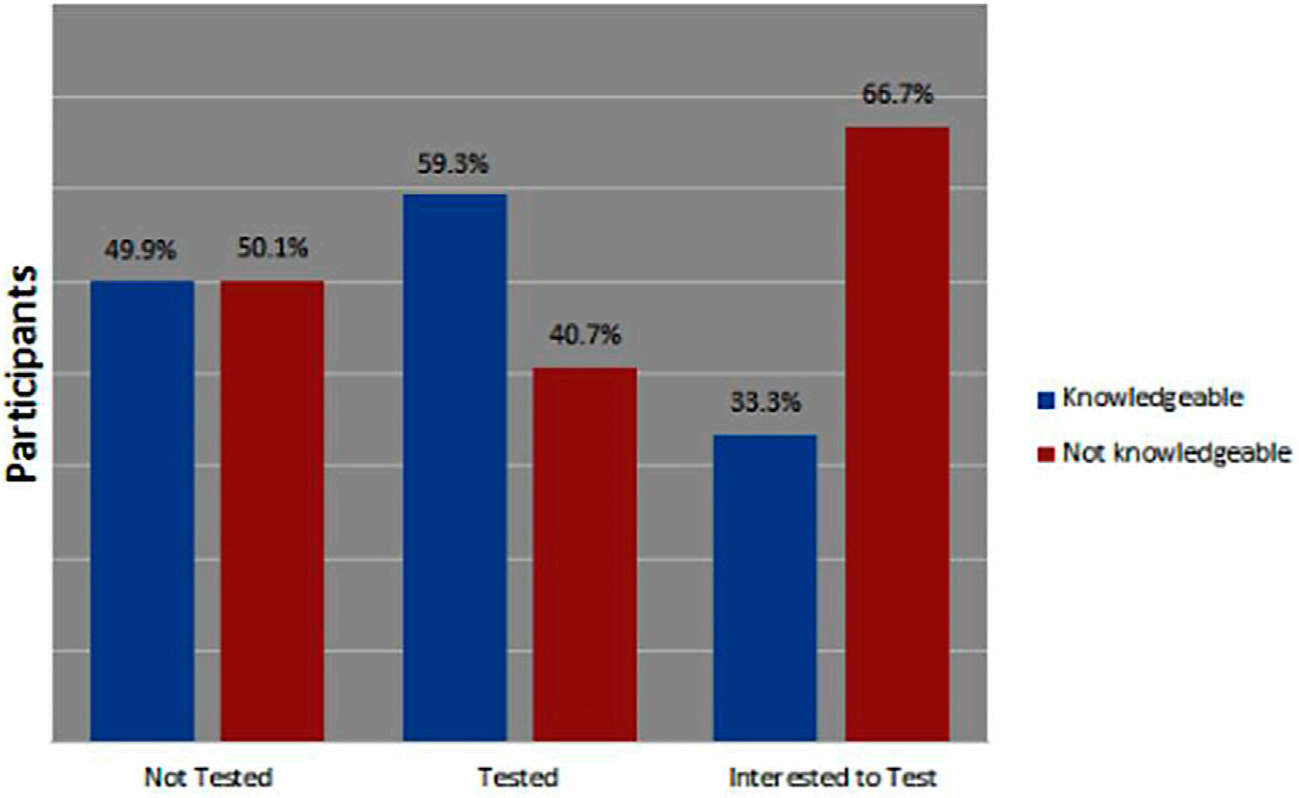
Knowledge about COVID-19 symptoms in Ondo state, Nigeria April-June 2021.

**FIGURE 2:**
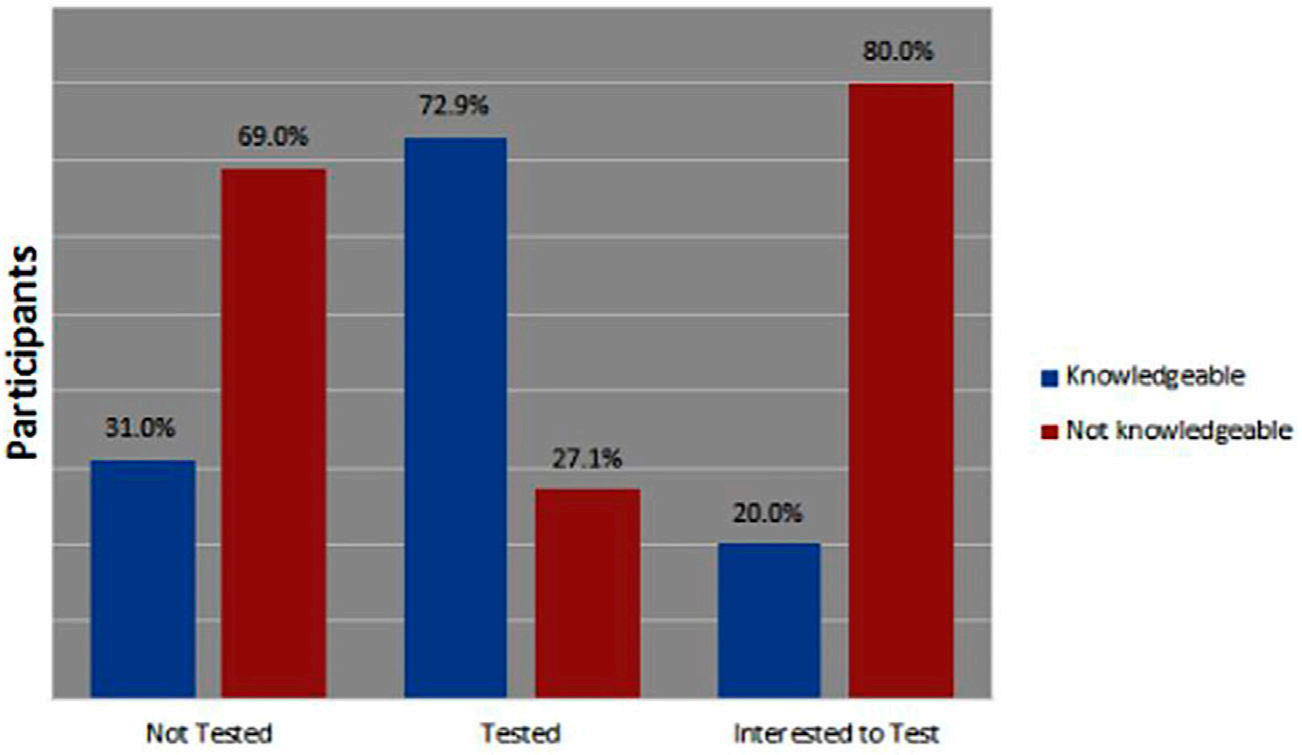
Knowledge about COVID-19 symptoms in Lagos state, Nigeria April-June 2021.

From Figures 1, 2 below, 59% (*n* = 16) of those who tested from Ondo state were aware about COVID-19 symptoms while 73% (*n* = 35) of those who tested in Lagos were aware about the COVID-19 symptoms.

### Attitudes Towards COVID-19 Testing

With regards to the attitudes, 6.4% (*n* = 45) of participants from the two states indicated that they had not been tested but are willing to test for COVID-19. Furthermore, fewer participants 1.3% (*n* = 9) reported that they were turned away from the testing centers due to being asymptomatic, low funds and payment for COVID-19 test, with Lagos state taking the whole population (Supplementary Table S1).

### Tests of Association

In addition to descriptive statistics, we also conducted chi-square tests of association as inferential analysis. In Lagos, the only socio-demographic factors that were significantly associated with testing and willingness to test at 5% significance level was education level (*χ* = 47.23, df = 10, *p* < 0.001). In Ondo, no socio-demographic factors were significantly associated with testing and willingness to test at 5% significance level Table 4.

Further investigation checking the association between testing and willingness to test in the two states revealed that awareness of nearby COVID-19 testing centers (*χ* = 94.41, df = 2, *p* < 0.001), internet access (*χ* = 11.03, df = 4, *p* = 0.03), access to technology devices (*χ* = 38.12, df = 6, *p* < 0.001), number of technology devices one has access to (*χ* = 25.88, df = 4, *p* < 0.001), and having another member of the family testing positive (*χ* = 33.83, df = 2, *p* < 0.001) were strongly associated with participants testing patterns in Lagos’. Similar investigations conducted in Ondo revealed that awareness of nearby COVID-19 testing centres (*χ* = 50.20, df = 2, *p* < 0.001), and having another member of the family testing positive (*χ* = 104.30, df = 2, *p* < 0.001) were strongly associated with participant’s testing patterns.

Finally, tests of association on participant’s knowledge, testing and willingness revealed that knowledge on preexisting conditions (*χ* = 39.89, df = 2, *p* < 0.001), symptoms (*χ* = 33.98, df = 2, *p* < 0.001), and where to go when having symptoms (*χ* = 22.99, df = 2, *p* < 0.001) were strongly associated with participants testing pattern in Lagos. In Ondo, however, only knowledge on preexisting conditions (*χ* = 10.20, df = 2, *p* = 0.01), and knowing where to go when having symptoms (*χ* = 6.55, df = 2, *p* = 0.03) were strongly associated with participant’s testing patterns, as shown on Table 4.

## Discussion

This study undertakes an in-depth look into the factors affecting individual testing for COVID-19, cutting across two states in south western Nigeria, Lagos state being the epicenter of the disease and Ondo state having relatively low prevalence. Results revealed that very few of the participants were willing to be tested for COVID-19 in both states. The small proportion of willingness exhibits great disparity with the results of Ukwenya [10] which revealed a great level of willingness to be tested among participants in Ondo state. The disparity between our findings and that of Ukwenya [10] could be as a result of a difference in study population. While our study population involved a mixture of urban, semi-urban and rural areas in both states, Ukwenya’s study population covered Akure, an urban area in Ondo state. Furthermore, our study was conducted at a period of reduced positive cases in Lagos and Ondo, when the wave of COVID-19 was flattening. Our results revealed the need to promote willingness for COVID-19 tests nationwide, excluding no one, as participants showed little concern in knowing their health status especially among rural dwellers. This is important as such knowledge will allow them to 1) rapidly eliminate uncertainty concerning their COVID-19-related health status; 2) enjoy healthcare benefits at an early stage if infected, thereby increasing the chances of a quick recovery; 3) actively prevent infecting others within their immediate environment, such as family and friends, as well as contacts in other relevant settings (work, school, church) and 4) obtain proof of no active infection, e.g., to avoid a quarantine when traveling internationally or having been in contact with an active infection case [11].

The age group 30–49 had the highest percentage of people who reported that they had tested and were also interested in testing for COVID-19 in Lagos and Ondo state. This interest in testing could be attributed to the mobile nature of people in this age bracket, coupled with compulsory testing imposed on members in this age group by reason of their places of work and businesses. People in this age bracket also constitute a higher percentage of the work force in Nigeria [12]. However, the group with the larger set of participants not willing to test were aged 29 and below, this could be because members of these age group have been reported to be less susceptible to coming down with severe COVID-19, with majority of the people of this age bracket showing mild symptoms or being asymptomatic [13]. This peculiarity, hampers their interest to test. Individuals from 60 years and above showed the least values for being tested or showing willingness to test. There was no significant association between Gender, marital status and COVID-19 testing behaviour. There was, however, a significant association between education levels and testing behaviour in Lagos, with the major percentage of those who reported to have tested being from tertiary institutions, furthermore, the same category had more people who were willing to test. Ondo state also showed similar pattern of association. This could be as a result of improved knowledge and better exposure, to information which could encourage good health seeking practices [8].

Our study showed that internet access as well as possession of a technological device, had a significant association with testing behaviour. Majority of the respondents reported having access to the internet at least once in a day and also possessing more than one device. More people who stated that they had done a COVID-19 test, also stated that they had access to the internet at least once in a day. The internet has become a major source of information hence, access to the internet can also be a factor for improving knowledge which could drive awareness and encourage testing [14]. However, there were still a lot of respondents who had access to the internet and yet were not interested in testing, which could also be as a result of the abundance of false news and wrong ideologies concerning the COVID-19 disease, that was being spread during the pandemic, with the internet used as a tool for such propagation [14].

With the advent of the COVID-19 infection, several underlying medical conditions were being discovered to be associated with COVID-19 leading to complications and resulting in mortality in most cases [15, 16]. This study revealed that a higher proportion of the population that tested in Lagos and in Ondo state were aware of the predisposing factors that increase the risk of contracting COVID-19, as well as the symptoms associated with COVID-19. Among those reporting not to have tested, the majority of them had no knowledge of COVID-19 predisposing factors as well as its symptoms. This shows that knowledge levels of a population about COVID-19 symptoms also affects the willingness to test in that population, these findings are similar to studies by Akwa and his colleagues [17, 18]. Akwa, in his study, reported that more than 80% of respondents had a good knowledge of the symptoms of COVID-19 which influenced their willingness to test. Hence, it can be inferred that knowledge about the symptoms and risk factors need to be encouraged to improve testing and curb the spread of COVID-19 disease.

Majority of Participants who reported that they had family members who tested positive for COVID-19, had also either tested or were willing to test. The result shows that having a relative or a close contact test positive for COVID-19 could encourage one to get tested. Another factor identified was awareness of testing centers, knowing where to get a COVID-19 test done also imparts testing behaviour. A large number of participants who had not tested for COVID-19 were also not aware of a testing center. The case was different for those who had tested or even those willing to test, as a good number of them were aware of a testing center. This finding was quite similar to a study carried out in Cameroon by Akomoneh [19] who reported that the majority of participants in his study either did not have testing sites or were not aware of a testing site. In major parts of Africa, especially Nigeria, where testing centers are not readily accessible in all areas, distance and time taken to reach a testing center can also pose a threat to an individual’s willingness to test. Our findings showed that a larger amount of people was situated within 5km-10km of the nearest testing center in Lagos and more than 10 km in Ondo state, however, majority of the participants who had tested or were willing to test for COVID-19 were situated less than 5km from a testing center in both states. The distance from the nearest testing center could be one of the reasons for low patronage in Lagos state and much more in Ondo. The low testing numbers can be attributed to the fact that there were not enough testing centers in the country, making access quite difficult for a larger percentage of people, especially those living in rural areas [20, 21]. Few respondents from Lagos State also reported that they have been turned away from a testing center citing reasons which includes; being asymptomatic, crowded centers and insufficient funds to transport themselves to the testing centers. On the contrary, this was not evident among participants in Ondo state with no case of reporting being turned away from any testing center.

Overall, the study highlighted, Education levels, family members who tested positive, awareness of testing centers, computer and internet access, as well as knowledge of pre-existing conditions and symptoms associated with COVID-19 as factors that could affect COVID-19 testing behaviour.

### Limitations

This study undertook simple random sampling, investigating the factors affecting testing behaviours in two states in the south western region of Nigeria, this may not represent the expected overall characteristics of the Nigerian population and, therefore, they do not represent the testing behaviors of the entire population. A wider approach with incorporation of more States representative of the six geopolitical zones of Nigeria should be taken. The survey was also taken at a time where COVID-19 cases had experienced significant reduction, this could lead to a reduction in urgency and the need to take proper health care measures amongst the study participants.

### Conclusion

Testing behaviors are affected by different social and environmental parameters, with knowledge of the causes, symptoms, predisposing factors, where one can get tested, as well as availability of testing centers nearby also being implicated as factors which is key in encouraging testing in this setting. In order to reduce the disparity of testing knowledge generally, more efforts should be put in enlightening and creating awareness about the symptoms and risk factors by necessary health information bodies and provision of more testing facilities should be made a great priority. This needs to be encouraged in order to improve testing and therefore curb the spread of COVID-19 infection.
