# Supplementary material for: Factors Affecting COVID-19 Testing Behaviours Among the Population in South Western Nigeria
Source: Int J Public Health. 2022 Oct 6;67:1604993. doi: 10.3389/ijph.2022.1604993 (PMC9582156; doi:10.3389/ijph.2022.1604993)
Supplement: Supplementary file 1 [file Table1.DOCX]

***Table S1: Attitudes towards COVID-1, Nigeria, April-June 2021***

| ***Variable*** | ***Lagos*** | ***Ondo*** |
| --- | --- | --- |
| ***Wish to test*** | | |
| *No* | 74.6%(n=229) | 89.4%(n=355) |
| *Yes* | 9.8%(n=30) | 3.8%(n=15) |
| *Not Applicable* | 15.6%(n=48) | 6.8%(n=27) |
| ***Turned away*** |  |  |
| *No* | 97.1%(n=298) | 100.0%(n=397) |
| *Yes* | 2.9%(n=9) | 0.0% (n=0) |
| ***Why turned away*** |  |  |
| *No symptoms* | 0.3%(n=1) | 0.0% (n=0) |
| *Crowd* | 0.3%(n=1) | 0.0% (n=0) |
| *Fund* | 0.3%(n=1) | 0.0% (n=0) |
| *Showing symptoms* | 0.3%(n=1) | 0.0% (n=0) |
| *Payment* | 0.3%(n=1) | 0.0% (n=0) |
| *Transferred* | 0.3%(n=1) | 0.0% (n=0) |
| *It’s #50,000* | 0.3%(n=1) | 0.0% (n=0) |
| *Shouldn’t sent people back* | 0.3%(n=1) | 0.0% (n=0) |
| *Having fever* | 0.3%(n=1) | 0.0% (n=0) |
